# Technical Comparison of Treatment Efficiency of Magnetic Resonance-Guided Focused Ultrasound Thalamotomy and Pallidotomy in Skull Density Ratio-Matched Patient Cohorts

**DOI:** 10.3389/fneur.2021.808810

**Published:** 2022-01-21

**Authors:** Abdul-Kareem Ahmed, Sijia Guo, Nathaniel Kelm, Ryan Clanton, Elias R. Melhem, Rao P. Gullapalli, Alexander Ksendzovsky, Howard M. Eisenberg, Timothy R. Miller, Dheeraj Gandhi

**Affiliations:** ^1^Department of Neurosurgery, University of Maryland School of Medicine, Baltimore, MD, United States; ^2^Department of Diagnostic Radiology and Nuclear Medicine, University of Maryland School of Medicine, Baltimore, MD, United States; ^3^Insightec, Tirat Carmel, Israel

**Keywords:** focused ultrasound (MRgFUS), thalamotomy pallidotomy, movement disorders, stereotactic ablation, skull density ratio

## Abstract

**Objective:**

MR-guided focused ultrasound (MRgFUS) is increasingly being used to treat patients with essential tremor (ET) and Parkinson's disease (PD) with thalamotomy and pallidotomy, respectively. Pallidotomy is performed off-center within the cranium compared to thalamotomy and may present challenges to therapeutic lesioning due to this location. However, the impact of target location on treatment efficiency and ability to create therapeutic lesions has not been studied. This study aimed to compare the physical efficiency of MRgFUS thalamotomy and pallidotomy.

**Methods:**

Treatment characteristics were compared between patients treated with thalamotomy (*n* = 20) or pallidotomy (*n* = 20), matched by skull density ratios (SDR). Aspects of treatment efficiency were compared between these groups. Demographic and comparative statistics were conducted to assess these differences. Acoustic field simulations were performed to compare and validate the simulated temperature profile for VIM and GPi ablation.

**Results:**

Lower SDR values were associated with greater energy requirement for thalamotomy (R^2^ = 0.197, *p* = 0.049) and pallidotomy (R^2^ = 0.342, *p* = 0.007). The impact of low SDR on efficiency reduction was greater for pallidotomy, approaching significance (*p* = 0.061). A nearly two-fold increase in energy was needed to reach 50°C in pallidotomy (10.9kJ) than in thalamotomy (5.7kJ), (*p* = 0.002). Despite lower energy requirement, the maximum average temperature reached was higher in thalamotomy (56.7°C) than in pallidotomy (55.0°C), (*p* = 0.017). Mean incident angle of acoustic beams was lesser in thalamotomy (12.7°) than in pallidotomy (18.6°), (p < 0.001). For all patients, a lesser mean incident angle correlated with a higher maximum average temperature reached (R^2^ = 0.124, *p* = 0.026), and less energy needed to reach 50°C (R^2^=0.134, *p* = 0.020). Greater skull thickness was associated with a higher maximum energy for a single sonication for thalamotomy (R^2^ = 0.206, *p* = 0.045) and pallidotomy (R^2^ = 0.403, *p* = 0.003). An acoustic and temperature field simulation validated similar findings for thalamotomy and pallidotomy in a single patient.

**Conclusion:**

The centrally located VIM offers a more efficient location for therapeutic lesioning compared to GPi pallidotomy in SDR matched cohort of patients. The impact on therapeutic lesioning with lower SDR may be greater for pallidotomy patients. As newer off-center targets are investigated, these findings can inform patient selection and treatment requirements for lesion production.

## Introduction

Magnetic resonance imaging-guided focused ultrasound (MRgFUS) is a promising, non-invasive technology that is being increasingly applied to treat various neurological disorders, including essential tremor and Parkinson's disease (1). However, all focused ultrasound neurological applications must overcome the physical limitations of the human skull, which historically required a craniectomy to enable ultrasound beams to reach the target (2–4). In 2002, Clement and Hynynen introduced an approach utilizing computer tomography data of the subject's calvarium to focus individual ultrasound beams through the intact skull (5). This transcranial technique works by registering CT data with MR imaging to predict the phase aberration and beam attenuation occurring at the calvarial-soft tissue interface, which is then corrected for by steering of individual transducer elements (6).

However, the efficiency of transcranial ablation using a hemispherical array of transducers is known to vary by target location, with a small treatment envelope being present in the center of the brain. In this envelope, the incident angles of individual transducer elements at the calvarium are optimal for current mid-frequency systems (7). As one targets locations farther from the brain's center, the incident angles increase, resulting in more beam deformity and overall lower treatment efficiency (7, 8). The efficiency of acoustic penetration through the intact cranium also varies by the skull density ratio (SDR). SDR is the median ratio at element points of cancellous to cortical bone in the calvarium, and ranges from 0 to 1, with a cutoff of around 0.4 considered to be more efficacious and used in the United States (9, 10). Unfortunately, in a review of 163 patients presenting to a single emergency room, 37 percent have an SDR below 0.4 and so are ineligible for treatment (10). Though it is known that a treatment envelope exists, that targets at the center of the brain are easier to lesion, the magnitude of variation in treatment efficiency between different locations within the treatment envelope and beyond, with different SDRs, has not been well delineated (11). This variation could have implications for new targets and for patients with low SDRs.

We therefore elected to compare the physical efficiency of MRgFUS ablation of the near-center thalamic ventral intermediate (VIM) nucleus for treatment of essential tremor (ET) to that of ablation of the more laterally and anteriorly located globus pallidus internus (GPi) for dyskinesias or motor fluctuations of Parkinson's disease (PD). Data were collected from individual cases performed at our institution, and partnering institutions, and matched by patients' skull density ratio (SDR), as SDR is the most significant determinant of treatment efficiency (9, 10). Acoustic and thermal simulations were performed to compare temperature profiles of each lesion target.

## Methods

### Patient Selection

To compare treatment characteristics, 20 patients treated with MRgFUS unilateral VIM thalamotomy for ET and 20 patients treated with unilateral GPi pallidotomy for PD were selected in pairs with matching SDRs, defined as values within 0.02 of each other. All 20 of 20 PD patients with available data were included, and 20 ET patients with similar SDRs were manually selected from a larger repository of treated patients. Patients were treated using the ExAblate 4,000 mid-frequency (670 kHz) head transducer system (InSightec, Haifa, Israel). All thalamotomies and 13 of the pallidotomies were performed in an identical fashion with the same neurosurgeon (H.M.E.), with low-level sonications to start for correction and alignment of the focal spot, followed by a relatively rapid rises in power to achieve ablative temperatures. The remaining pallidotomies were performed at partnering institutions. Parkinson's disease patients were treated in a prospective, open-label, multicenter trial of unilateral MRgFUS ablation of globus pallidus interna (NCT02263885). Institutional review board (IRB) approval was obtained at respective institutions and anonymized data were available in accordance with a prior data sharing agreement (NCT03100474). ET patients were all treated at the University of Maryland, Baltimore, and their data were entered into a prospectively maintained IRB-approved database (NCT01827904; NCT02289560). All data were anonymized.

### Data Collection

**Figure 1:**
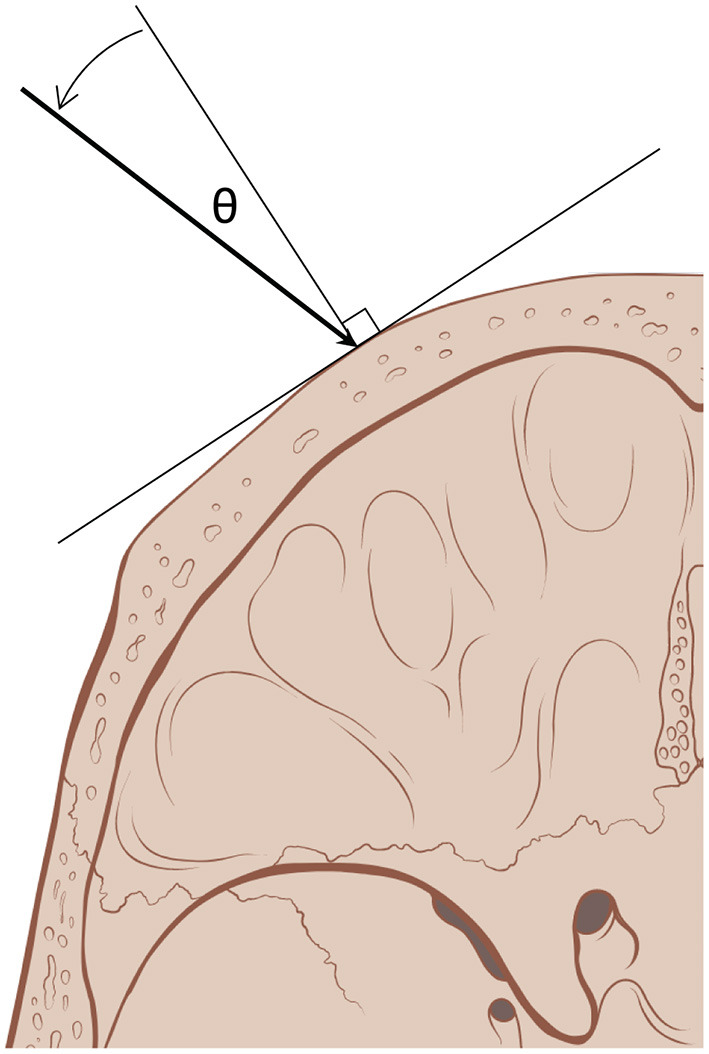
Incident angle (θ) measures the degree from normal, orthogonal, at which acoustic beams (arrow) emitted by ultrasound elements reach the outer table of the skull. With a smaller incident angle, a greater proportion of the beam's energy traverses the skull (skull art reused with permission from Patrick J. Lynch and C. Carl Jaffe, MD).

Data on patient disease, age, and sex were collected. SDR, mean skull thickness, and skull surface are data were downloaded from respective ultrasound systems. To evaluate aspects of treatment efficiency, the incident angles of acoustic beams (θ) on the outer table of the skull emitted by ultrasound elements, the number of sonications for treatment, the sonication time (minutes), the maximum average temperature reached (°C, T_max_), the maximum energy for a single sonication (kilojoules, kJ), and the energy required to reach a temperature of 50 °C (kJ) were recorded (Figure 1). These were compared between thalamotomy and pallidotomy. Given that the treatment efficiency and not clinical efficacy was the objective of this study, and the fact that clinical outcomes for the two treatment populations would not be congruent, we did not collect or compare clinical outcomes. However, the clinical outcomes for the PD cohort has been published and this data is available (12).

### Statistical Analysis

Demographic and comparative statistics were conducted to assess differences between the treatment groups and to develop regression models to assess relationships between different parameters (independent samples *t*-test, Mann–Whitney *U* test, and Pearson's χ^2^ coefficient) (13). Energy to reach 50°C was calculated using logarithmic fit curves of input energy compared to temperature rise to account for differences in treatment approaches between patients and operators. A univariate linear regression model of SDR and energy required to reach 50°C was developed for each treatment group and compared, and additional linear regression models were developed. A benchmark of 50°C was used as all treatments reached or exceeded this temperature. Normal distributions were assumed for variables, however not for skull thickness (10, 14). The alpha level was set to 0.05. All statistical tests were computed with IBM SPSS Statistics software, version 23.0 (Armonk, NY, *IBM Corp*).

### Acoustic and Temperature Field Simulation

To better understand the differences of lesion formation between MRgFUS thalamotomy and pallidotomy, acoustic field simulations were performed to evaluate the resulting acoustic profile. The acoustic fields within the head were simulated using a 3-D finite differences algorithm, which aims to solve the full Westervelt equation, a method to estimate temperature rise through heterogenous tissues (15). The acoustic properties of the skull were modeled based on CT images of one treated ET patient (16). Temperature simulation was estimated using the inhomogeneous Pennes equation of heat conduction (17). By solving the bio-heat equation with the calculated acoustic intensity field as the input, peak tissue temperature distribution was calculated. Both acoustic and temperature simulations were done at a resolution of 1 × 1 × 3 millimeters, to match the resolution of MR thermometry images obtained during treatment. The resulting simulated temperature profile for VIM and GPi ablation was compared on the same patient, particularly, the VIM temperature profile was compared with the MR thermometry data acquired during the treatment.

## Results

Data on 40 patients were collected and analyzed (Table 1). Patients treated with thalamotomy (*n* = 20) were older than patients treated with pallidotomy (*n* = 20) (*p* < 0.001). The proportions of patients in each treatment group did not differ by sex (*p* = 0.490). The mean SDR for thalamotomy was 0.54 (SD: 0.071) and for pallidotomy was 0.55 (SD: 0.067), (t-test, *p* = 0.584). The mean of absolute differences in SDR between treatment groups was 0.013 (SD: 0.0092). The mean skull thickness, an average of thickness calculated at every element point across the skull area treated, was 6.1 mm (SD: 1.1) for thalamotomy and 6.4 mm (SD: 1.1) for pallidotomy. The distribution of skull thickness between these groups was not different (Mann-Whitney *U* test, *p* = 0.327). There was no difference in mean skull surface area between thalamotomy, 340.3 cm^2^ (SD: 30.2), and pallidotomy, 336.7 cm^2^ (SD: 22.9), (*t*-test, *p* = 0.678).

**Table 1:** Demographics, skull parameters, and treatment characteristics of magnetic resonance-guided focused ultrasound unilateral thalamotomy and pallidotomy.

	**Thalamotomy**	**Pallidotomy**	***p***
**Demographics**			
Patients (*n*)	20	20	n/a
Mean age, years (SD)	70.4 (8.4)	56.3 (11.2)	<0.001
Sex ratio (M:F)	15:5	13:7	0.490
**Skull parameters, mean**			
Skull density ratio (SD)	0.54 (0.071)	0.55 (0.067)	0.584
Skull thickness, mm (SD)	6.1 (1.1)	6.4 (1.1)	0.327^a^
Skull surface area, cm^2^ (SD)	340.3 (30.2)	336.7 (22.9)	0.678
**Treatment characteristics, mean**			
Incident angle, θ, (SD)	12.7 (1.1)	18.6 (1.5)	<0.001
Elements with incident angles <25° (SD)	982.4 (21.9)	791.7 (89.0)	<0.001
Sonications (SD)	18.0 (7.3)	15.8 (3.0)	0.233
Sonication time, min, (SD)	110.4 (53.5)	104.9 (26.1)	0.679
Maximum average temperature, °C, (SD)	56.7 (2.2)	55.0 (2.1)	0.017
Maximum energy, kJ (SD)	12.4 (6.3)	16.6 (7.8)	0.069
Energy, kJ, to 50°C (SD)	5.7 (2.8)	10.9 (6.5)	0.002

*mm, millimeters; min, minutes; SD, standard deviation; kJ, kilojoules; M, male; F, female; R, right; L, left*.

*p-value: independent samples t-test. Mann–Whitney U test, or Pearson's χ^2^ coefficient*.

a *Mann–Whitney U test, reflects comparison of populations, not means*.

**Figure 2:**
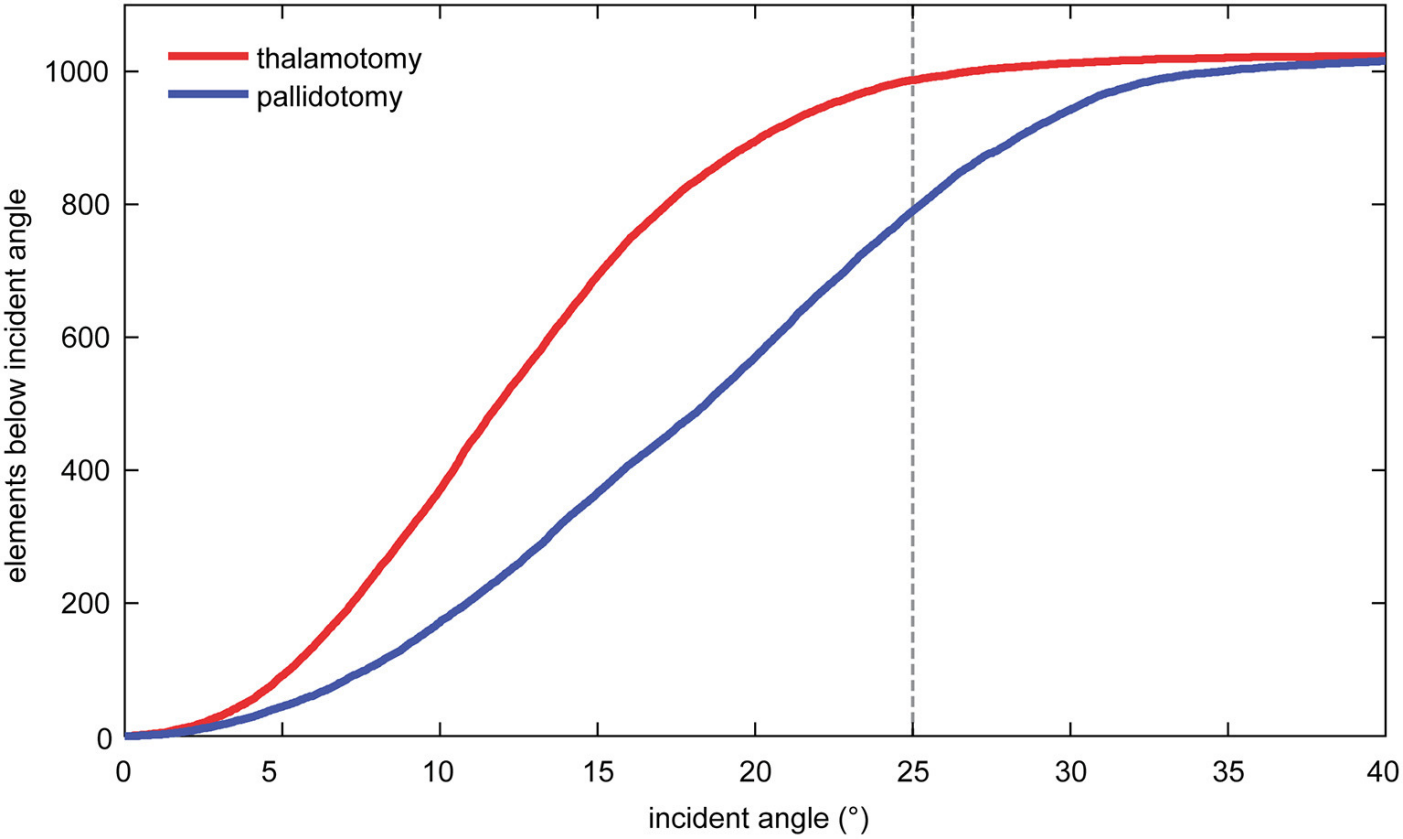
The mean number of elements that emitted beams with incident angles <25 degrees was greater for thalamotomy of the ventral intermediate nucleus (VIM) than for pallidotomy of the globus pallidus internus (GPi).

The mean incident angle of acoustic beams was less in thalamotomy (12.7°, SD: 1.1) than in pallidotomy (18.6°, SD: 1.5), (*t*-test, *p* < 0.001). The mean number of elements of 1,024 maximum elements that emitted beams with incident angles <25 degrees, previously shown to be an optimal incident angle, was greater for thalamotomy (982.4, SD: 21.9) than for pallidotomy (791.7, SD: 89.0) (*t*-test, *p* < 0.001) (Figure 2) (1, 18).

Treatment groups did not differ in the mean number of sonications (*p* = 0.233) or the mean sonication time (*p* = 0.679). The mean maximum average temperatures reached was higher in the thalamotomy group (56.7°C, SD: 2.2) than in the pallidotomy (55.0°C, SD: 2.1), (*t*-test, *p* = 0.017). Mean maximum energy for a single sonication was higher in pallidotomy (16.6 kJ, SD: 7.8) than in thalamotomy (12.4 kJ, SD: 6.3), approaching significance (*t*-test, *p* = 0.069). The mean energy needed to reach 50 °C was nearly two-fold higher in pallidotomy (10.9 kJ, SD: 6.5) than in thalamotomy (5.7 kJ, SD: 2.8), (*t*-test, *p* = 0.002).

**Figure 3:**
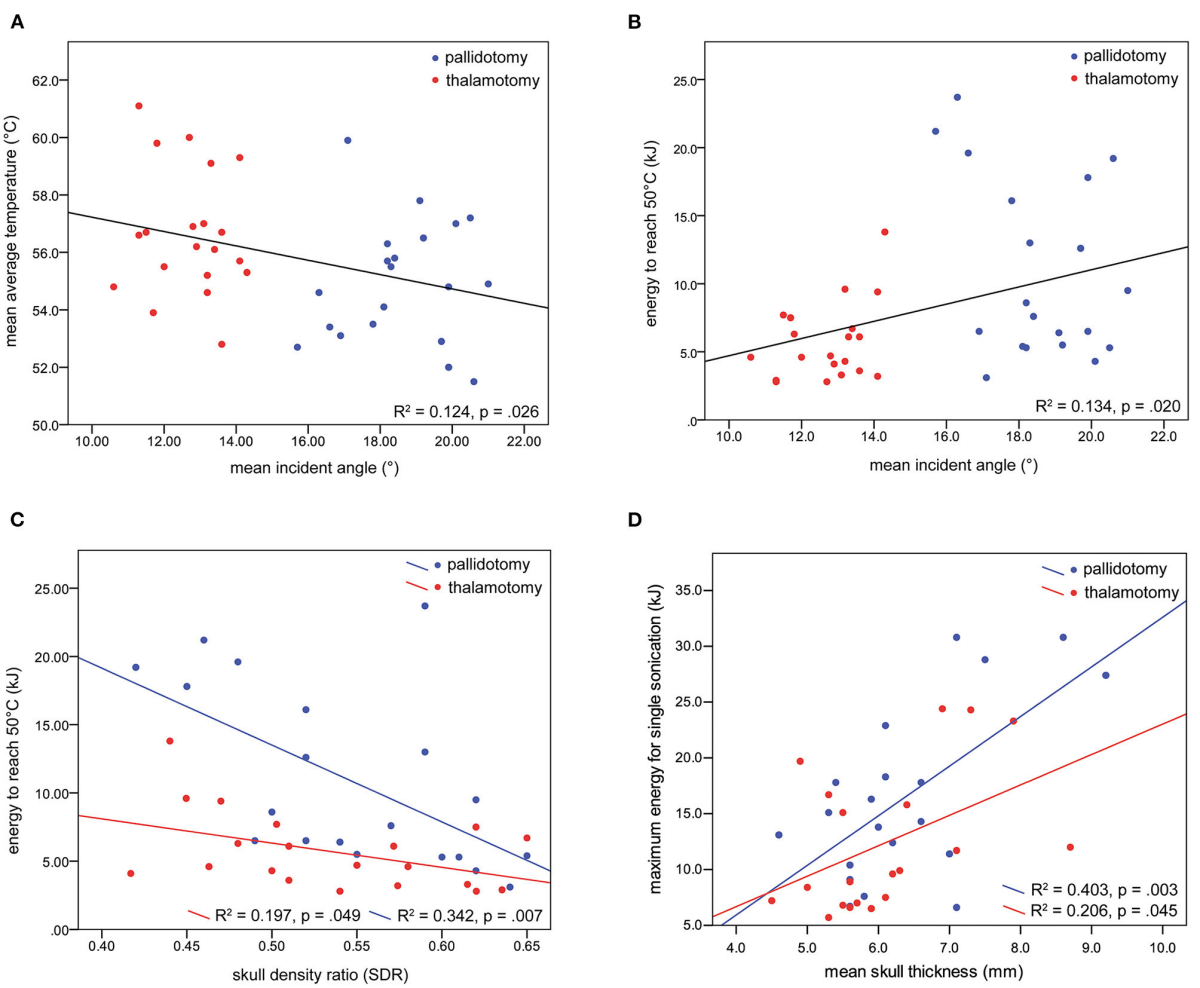
Univariate regression models were developed. A lesser mean incident angle was associated with a higher maximum average temperature reached **(A)**, and less energy needed to reach 50 °C **(B)**. A lower SDR was correlated with more energy needed to reach 50 °C, for thalamotomy and for pallidotomy, nearly to a greater degree for pallidotomy **(C)**. Greater skull thickness was associated with greater energy for a single sonication **(D)**.

Univariate linear regression models were developed to determine the relationships between mean incident angle and treatment characteristics, and SDR and treatment characteristics. For all patients, a lesser mean incident angle was associated with a higher maximum average temperature reached (slope = −0.250, R^2^ = 0.124, *p* = 0.026), and less energy needed to reach 50 °C (slope = 0.632, R^2^ = 0.134, *p* = 0.020) (Figures 3A,B). A lower SDR was correlated with more energy needed to reach 50 °C, more strongly for thalamotomy (slope = −17.8, R^2^ = 0.197, *p* = 0.049) than for pallidotomy (slope = −56.3, R^2^ = 0.342, *p* = 0.007). The slope of this relationship was steeper for pallidotomy, approaching significance on comparison between thalamotomy and pallidotomy (*p* = 0.061) (Figure 3C).

A greater skull thickness was associated with more energy needed to reach 50°C among all patients, however analysis by treatment revealed this held true for pallidotomy, not for thalamotomy, (slope = 3.3, R^2^ = 0.324, *p* = 0.009). Additionally, a greater skull thickness was associated with a higher maximum energy for a single sonication for thalamotomy (slope = 2.7, R^2^ = 0.206, *p* = 0.045) and pallidotomy (slope = 4.4, R^2^ = 0.403, *p* = 0.003), but the slopes of these relationships did not differ (*p* = 0.345) (Figure 3D). Skull thickness was not associated with maximum average temperature reached. Skull surface area was not associated with any measure of treatment efficiency. In a multiple regression model, incident angle (slope = 0.688, *p* = 0.002), SDR (slope = −29.4, *p* = 0.004), and skull thickness (slope = 2.4, *p* < 0.001) correlated with energy needed to reach 50 °C (F(3, 36) = 12.719, *p* < 0.001, R^2^ = 0.515). Age and sex did not correlate with each other, or with SDR, incident angle, maximum average temperature reached, or energy needed to reach 50 °C.

**Figure 4:**
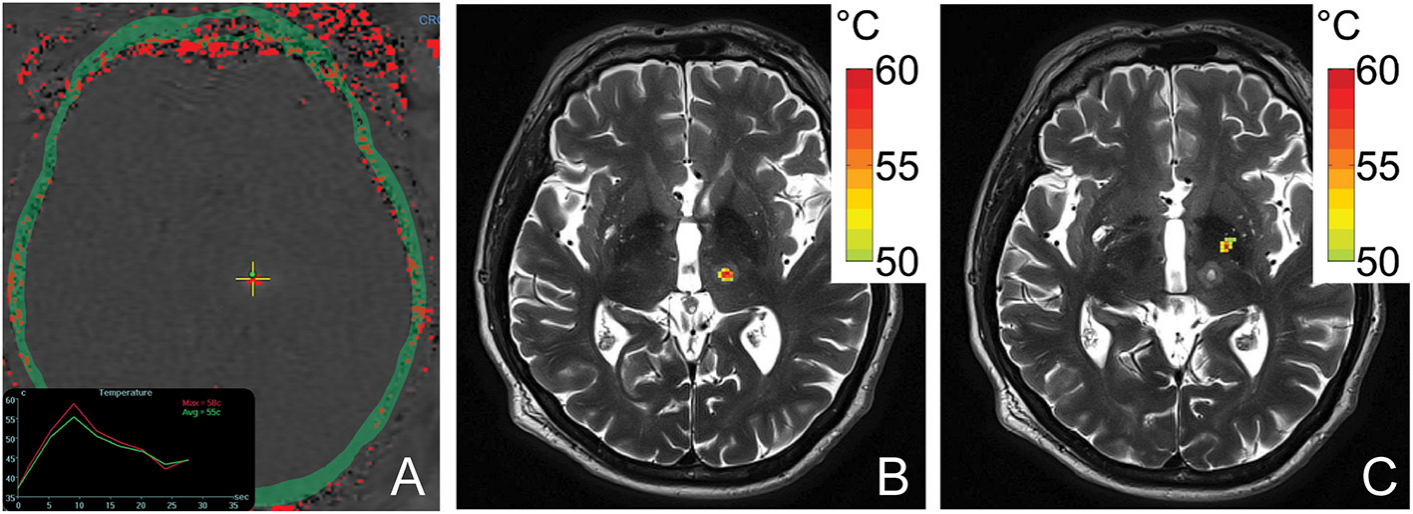
An MR thermometry image acquired during the treatment of ET with focused ultrasound thalamotomy showing 58°C was achieved as the maximum temperature on this patient **(A)**. An example of temperature simulation results is shown when targeting VIM **(B)** and GPi **(C)** on the same patient. The temperature fields were registered to the post 1-day T2-weighted images. The globus pallidus internus lies antero-lateral compared to the ventral intermediate nucleus.

Simulated temperature maps for both VIM (Figure 4B) and GPi (Figure 4C) ablation on a single ET patient with the same sonication duration and power were rendered. The estimated peak temperature was 59.8 °C and 57.8 °C at the VIM and GPi targets, respectively. On the VIM target, the simulated peak temperature was quite close to the peak temperature (59.8 °C) recorded by MR thermometry (Figure 4A) during the treatment under the same conditions.

## Discussion

Our results demonstrate that MRgFUS ablation of the GPi is less efficient than ablation of the thalamic VIM nucleus in patient populations with matched skull density ratios. The energy required to reach 50 °C during pallidotomy was nearly twice that of thalamotomy. This is likely due to the greater incident angles of individual ultrasound elements when targeting the more antero-laterally located GPi, which in turn results in decreased transmission and greater reflection of acoustic energy at the skull outer table (Figure 4) (1, 18, 19). This reduced transmission of acoustic energy also likely explains the lower maximum average temperature achieved during GPi ablation although, in this present cohort of patients, definitive evidence of successful lesioning was always observed and confirmed on post-operative MRI.

Similar to prior studies, SDR and skull thickness significantly influenced treatment efficiency at both targets in our cohort, SDR more so than skull thickness (9, 10, 19). However, there was a strong trend toward a greater influence of lower SDR on pallidotomy efficiency. Greater skull thickness also uniquely reduced efficiency in pallidotomy patients. This again may be due to the location of the GPi in relation to the cranium. While prior authors have demonstrated that VIM ablation may still be successfully performed in low-SDR candidates, our results suggest that operators should be more cautious when proceeding with pallidotomy in this patient population (10, 20). Yet, it is important to note that although lower SDRs have been correlated with a greater energy requirements for MRgFUS ablation, they have not been shown to impact clinical outcomes (10, 21). Further investigation regarding the feasibility of MRgFUS pallidotomy in low SDR patients is warranted. Future innovations in treatment algorithms could focus on selectively turning off elements with extreme beam angles to increase or modify treatment efficiency, especially in patients with low SDR. Other therapeutic strategies in low SDR patients may rely on either repeated prolonged exposures of the intended target to lower than ideal temperatures to accomplish ablative lesioning or alternatively steeper ramp up of temperatures during treatment since repeated sonications with smaller energy increments may result in reduction in skull efficiency (22).

An acoustic and temperature field simulation demonstrated a higher peak temperature for thalamotomy than pallidotomy in the same ET patient, an internal control. The close estimated peak temperature by simulation compared with the treatment data indicated the accuracy of the simulation model. The simulated thermal profiles also closely resemble those observed in ET and PD treatment cases (22, 23). GPi lesions assume an elongated, ellipsoid shape extending in the inferolateral to superomedial direction due to the off-center target location which results a larger mean incident angle and therefore, an uneven energy distribution (22). These same factors result in an overall reduced peak acoustic intensity generated at the GPi compared to VIM, with resulting lower achievable peak ablation temperatures. In contradistinction, the near-center location of the VIM results in spherical lesions as well as, on average, a higher peak temperature under the same sonication conditions.

Although it was beyond the scope of the current study, there was no evidence to suggest that the lower treatment efficiency of pallidotomy impacted the ability to generate a lesion at the GPi in our cohort. Specifically, all Parkinson's patients included in the current investigation underwent successful GPi ablation with evidence of the expected T2 hyperintense, diffusion restricting lesion on post-procedure MRI (12). One potential explanation for how GPi ablation was achieved despite the significantly reduced treatment efficiency was our prior observation that pallidotomy may be accomplished using repetitive lower maximum average temperature sonications (22). Consequently, the peak temperature achieved in GPi ablation may be less important than the accumulative thermal dose delivered at this target. Moreover, GPi ablations were performed in the setting of a trial, with a cut-off SDR value of 0.4 (12). Therefore, although our data predicts that GPi of the patients with SDR <0.4 may be much more difficult to lesion compared to VIM, this is a speculation since we did not have any patients lower than 0.4 SDR in the trial.

In one attempt to perform MRgFUS lesioning of the hippocampus for mesial temporal lobe epilepsy, adequate temperatures could not be reached, likely secondary to the peripherality of the target (24). There are multiple efforts aimed at improving the treatment envelope in MRgFUS systems, as more lateral and peripheral targets are investigated for epilepsy, tumor ablation, and psychiatric diseases (24–27). Cadaveric phantom models treated with MRgFUS thermal ablation provide a method to test new target sites, and corresponding treatment requirements (28). In patients who undergo a craniotomy for initial treatment of a tumor, cranial prostheses optimized for acoustic penetration can be implanted instead of native bone to facilitate future MRgFUS treatment (29). Additionally, a cadaveric simulation study tested a patient-specific conformal array that uses concave ultrasound elements and pulsed ultrasound to improve energy delivery to peripheral targets (30). Furthermore, for targets near the skull base, a blocking algorithm to selectively exclude ultrasound elements can prevent heating of the skull and neurovascular damage (31, 32). Lastly, optimization of transcranial focusing, such as with echo-focusing, may further expand the treatment envelope and improve the treatment efficiency for MRgFUS, even in low SDR patients (33, 34).

This study has several important limitations, including its retrospective design and smaller cohort size; the latter was due to the limited number of pallidotomy patients with data available for analysis. Also, SDRs are specific between individuals, and were unable to be matched exactly between cohort groups, which may have influenced our results. Our data was not granular enough to determine the effect of local SDR on treatment efficiency, so we used average SDR as prior studies have done. Although most of our patients were treated at a single institution, differences in treatment practices between institutions can be difficult to parse out when grouping individuals treated for the same disease. Similarly, treatment practices and strategies can vary between teams affecting treatment duration. Finally, we did not include any patients with lower SDRs (i.e. <0.4), which would pose a significantly greater challenge for successful MRgFUS lesioning due to reduced treatment efficiency. Future studies should focus on this cohort of patients to understand the true impact of larger incident angles while treating off-center locations.

## Conclusion

MRgFUS thalamotomy of the VIM for essential tremor has higher treatment efficiency characteristics than pallidotomy of the GPi for Parkinson's disease. This is likely due to the central location of the VIM. As new off-center targets within the skull are investigated for MRgFUS thermal ablation and treatments are considered in low SDR subjects, these findings can inform appropriate patient selection and physical treatment requirements (35).

## Data Availability Statement

The raw data supporting the conclusions of this article will be made available by the authors, without undue reservation.

## Ethics Statement

The studies involving human participants were reviewed and approved by University of Maryland, Baltimore Institutional Review Board (Exemption HP-00093725). Written informed consent for participation was not required for this study in accordance with the national legislation and the institutional requirements.

## Author Contributions

A-KA organized the database. A-KA and TM performed the statistical analysis. A-KA, DG, and TM wrote the first draft of the manuscript. SG and AK wrote sections of the manuscript. All authors contributed to manuscript revision, conception, read, design of the study, and approved the submitted version.

## Conflict of Interest

Authors NK and RC were employed by company Insightec. HE and DG were on the advisory board of Insightec. The remaining authors declare that the research was conducted in the absence of any commercial or financial relationships that could be construed as a potential conflict of interest.

## Publisher's Note

All claims expressed in this article are solely those of the authors and do not necessarily represent those of their affiliated organizations, or those of the publisher, the editors and the reviewers. Any product that may be evaluated in this article, or claim that may be made by its manufacturer, is not guaranteed or endorsed by the publisher.
